# Rationalising a spectrum of problematic exercise: A qualitative study

**DOI:** 10.1177/13591053241274471

**Published:** 2024-09-13

**Authors:** Kate Nicholls, Jane Ogden

**Affiliations:** University of Surrey, UK

**Keywords:** exercise addiction, exercise motivation, problematic exercise, qualitative study

## Abstract

Whilst a substantial body of evidence supports the benefits of exercise for physical and mental health, the overfocus on the benefits of exercise could result in harmful behaviours in some individuals. Conceptualised as a behavioural addiction, research often dichotomises the behaviour through a medical diagnostic model. The present qualitative study explored the meaning of problematic behaviour from the exerciser’s perspective. Nineteen UK-based frequent exercisers were interviewed regarding their experiences. Thematic analysis described three themes: ‘relentlessly pushing the limits’ of their personal best and comparing to others; ‘an enabling community’ which could promote problematic behaviours; and ‘the complexity of the ideal body’ focussing on perceptions of weight maintenance. Transcending these themes was the notion of ‘rationalising choices’. The results indicate that participants felt that the benefits outweighed the costs, encouraging them to continue, even when causing harm. These findings support the notion of problematic exercise as a continuum, rather than dichotomous.

## Introduction

Regularly taking part in forms of exercise that focus on cardiovascular fitness and strength building has been shown to have a wide variety of positive physical, mental and social outcomes (Davis et al., 2015; Penedo and Dahn, 2005; Warburton et al., 2006). Exercise has long been encouraged as one of the most effective means of managing health (Sparling et al., 2000). Some exercisers, however, form a more problematic relationship with exercise, driving them to continue exercising despite negative consequences to themselves and others. Problematic exercise, often conceptualised as a behavioural addiction, can have negative consequences for work or study, social and personal relationships, and tends to lead to increased risk of injury and potentially overtraining syndrome or RED-S (Griffiths, 1997; Kreher and Schwartz, 2012; Langbein et al., 2021; Stellingwerff et al., 2021). Researchers have variously labelled this condition as exercise dependence, exercise addiction, problematic exercise, obligatory exercise or compulsive exercise (Blumenthal et al., 1984; Griffiths, 1997; Hausenblas and Downs, 2002; Kotbagi et al., 2017; Yates, 2013). Exercise addiction is the most commonly used term, typically based on the medical model of addiction, and includes tolerance, withdrawal, stereotyped behaviour, increasing salience, relapse, euphoria, mood modification, excessive time spent exercising, exercising more frequently or longer than intended and continuing to exercise despite subjective awareness of negative consequences (DE Coverley Veale, 1987; Griffiths, 1997; Hausenblas and Downs, 2002).

Much of the existing literature on addiction uses a-priori defined criteria and draws upon a dichotomous diagnostic model in line with DSM-5 (Baker et al., 2023). Further, addiction, by definition, results in negative consequences or harm to individuals (Szabo et al., 2015), and it has been argued that addiction criteria should be inherently negative (Griffiths, 2020). For behavioural addictions, however, this is often more complex. For example, whilst research exploring the experiences of problematic gamblers reflects some of the traditional criteria of addiction, such as compulsion, chasing losses, tolerance and the triggering impact of life events (Rogier et al., 2020; Rolando and Beccaria, 2019), for some, life events can be turning points towards recovery. Likewise, whilst mood modification is one of the criteria for exercise addiction in the Exercise Addiction Inventory (Griffiths et al., 2005), exercise can be used healthily as a mood modifier by those seeking relief from stress, depression or anxiety (Moreau et al., 2023), and is encouraged by health authorities as a self-help option (NHS, 2022a, 2022b).

Therefore, whilst exercise behaviour can become problematic, less is known about how those who frequently exercise conceptualise problematic behaviour. The current qualitative study therefore aimed to explore problematic exercise from the perspective of the exerciser. To avoid any preconceived ideas of problematic exercise, this study aimed to ask frequent exercisers for their experience of exercise, both positive and negative, as well as their interpretation of problematic exercise and exercise addiction. Although it is understood that frequency of exercise alone cannot define the problematic nature of exercise, it is also expected that exercise would not become problematic in individuals who exercise at, or less frequently than, the minimum weekly recommendation. This study therefore aimed to explore the negative consequences resulting from exercise, and the exercisers’ own perspectives of whether these consequences constitute problematic exercise.

## Method

### Design

The study used a qualitative design with in-depth interviews.

### Positionality

This study was positioned within critical realism. Through this approach it was acknowledged that individuals’ experiences would mediate their perception on the nature of their exercise; whether they understand their exercise to be problematic or not reflects their interpretation of reality. However, this study also acknowledges a more objective reality of medical consequences of continuing to exercise through injuries, such as muscle sprains or broken or dislocated bones. The researchers’ backgrounds also contributed to the development of the findings, with both researchers reflecting that they participate in frequent but not problematic exercise. Reflexivity was therefore integral to the analysis to interrogate interpretations of the interviews. The analysis was therefore informed by critical realism and whilst the researchers recognised some objectively problematic behaviour, the focus on the perspective of the exerciser, meant that an inductive approach to data analysis was important.

### Participants

Participants were invited to interview if they were aged 18 or over, lived in the UK and regularly engaged in more than 3 hours per week of exercise. These broad inclusion criteria aimed for diversity both in terms of demographics and type of exercise or sport participation. Twenty participants completed the consent form, with 1 found ineligible, resulting in 19 participants (11 females, 8 males), aged between 22 and 59 years. This number is equivalent to other thematic analysis studies and aligns with the recommendation from Braun et al. (2016). It was felt that the dataset had sufficient breadth and depth to allow for the development of themes across a spectrum of exercise experiences. The participants were predominantly white, well-educated and employed full-time. Most participants listed more than one regular physical activity, the most popular of which was running (*n* = 14), followed by yoga (*n* = 7) and weight training (*n* = 6).

Prior to the interview, participants completed the Exercise Dependence Questionnaire (EDQ) (Ogden, Veale and Summers, 1997) with scores ranging from 89 to 147 (*m* = 109.89, *SD* = 14.20). The EDQ does not have a specified cutoff value, however, some research has used a cut-off of 116 to distinguish between those with possible exercise dependence and those without (Bamber et al., 2000). Additionally, in pre-interview questions, none of the participants agreed that they felt exercise was ‘a problem’ instead stating, ‘definitely not’ (*n* = 10), ‘probably not’ (*n* = 7) or ‘may or may not’ (*n* = 2). During the interviews, three participants seemed to relate to the idea of exercise addiction, five were unsure or classified themselves as borderline addicted, two had recovered from a diagnosis of disordered eating and nine said that they did not have exercise addiction. Participant details can be found in Table 1, pseudonyms have been used for participants’ anonymity.

**Table 1.** Details of the interview participants.

Pseudonym	Age	Gender	Ethnicity	Education Level	Employment Status	Exercise routine	EDQ Score	‘My exercise is a problem’ response	Self-reported exercise addiction
Alison	48	Female	White	Master’s degree	Employed Full Time	4.5 hours per week (four runs, one conditioning class, daily walk)	103	Definitely Not	No
Andrew	42	Male	White	A Levels	Employed Full Time	About 4 hours per week (three runs, two weight training)	109	Definitely Not	No
Caitlin	31	Female	White	Doctorate	Employed Full Time	13.5 hours per week (six runs and cycle commute)	147	May or May Not	Maybe
Carlos	34	Male	Mixed Ethnic Background	Bachelor’s degree	Employed Full Time	7.5 hours per week (two runs and cycle commute)	98	Probably Not	No
Charlie	23	Male	White	Master’s degree	Student	7–8 hours per week (three weight training sessions, three yoga sessions and one run)	119	May or May Not	Yes
Chloe	26	Female	White	Bachelor’s degree	Employed Full Time	9 hours per week (two callisthenics sessions, three runs and yoga)	100	Definitely Not	No
Emma	27	Female	White	Master’s degree	Student	9 hours per week (three swims, two or three yoga or Pilates classes and daily walk)	100	Definitely Not	Recovered from Eating Disorder
Georgina	50	Female	White	Doctorate	Employed Full Time	7 hours per week (five runs, two spin classes)	104	Probably Not	Maybe
James	31	Male	White	Bachelor’s degree	Employed Full Time	9.5 hours per week (football/soccer and 2–4 gym sessions)	107	Definitely Not	Yes
Laura	24	Female	White	Master’s degree	Student & Part-time employment	4.5–6 hours per week (netball and swimming)	109	Probably Not	No
Louise	26	Female	White	Master’s degree	Student	6 hours per week (three runs, three gym sessions)	107	Definitely Not	No
Manpreet	36	Female	Asian/British Asian	Bachelor’s degree	Self Employed	7 hours per week (four weight training, 3 hours of walking)	108	Definitely Not	No
Marcus	23	Male	Mixed Ethnic Background	Master’s degree	Student	8–9 hours per week (weight training)	122	Probably Not	No
Martin	59	Male	White	Master’s degree	Employed Full Time	21 hours per week (daily run)	103	Definitely Not	No
Naomi	22	Female	White	Master’s degree	Employed Full Time	3 hours per week (three gym sessions including treadmill and weights, one dance/yoga class, walking)	89	Definitely Not	Recovered from Eating Disorder
Paul	34	Male	White	Doctorate	Employed Full Time	2–7 hours per week (three runs and 2/3 weight training sessions)	111	Probably Not	Yes
Richard	56	Male	White	Master’s degree	Employed Full Time	6-7 hours per week (two squash games, three yoga classes, one gymnastics class, daily run)	102	Probably Not	Maybe
Sophie	27	Female	White	Bachelor’s degree	Employed Full Time	14 hours per week (two runs, two cycles, one swim)	108	Definitely Not	Maybe
Zoe	29	Female	White	Doctorate	Employed Full Time	25 hours per week (eight classes, at least 5 hours rock climbing, one swim, daily run)	142	Probably Not	Maybe

### Procedure

After approval by the university’s ethics committee, UK-based adults who regularly engaged in exercise activities for over 3 hours per week were recruited online via social media and physical advertisements in gyms or other sports settings. Interested individuals were provided with an information sheet and consent form. No incentives were offered for participation. Given the potential for sensitive topics to arise, one-to-one interviews were conducted, allowing for a more comfortable and in-depth discussion. For convenience, interviews were conducted online at a mutually convenient time using an online meeting tool with the first author, a female PhD student. Interviews lasted approximately 30 minutes (*m* = 27 minutes 39 seconds, *SD* = 6 minutes 24 seconds). This was the time requirements set out in the participant information briefing document, as many of the participants were in full-time employment and were volunteering their time without incentives. The interviews were recorded (both audio and visual) and later transcribed verbatim by the first author.

### Interview schedule

The interviews were semi-structured to allow flexibility and enable participants to bring their own topics of importance to the conversation. Initially, the participants were introduced to the interviewer and informed that the research focus was on when exercise can become problematic for some people and understanding the positives and the consequences of exercise. Participants were told that they could end the interview at any time. The interview guide covered the participants’ typical exercise routines and their personal exercise history; their motivations for exercise, as well as the positive and negative aspects they experienced from their chosen exercise. Participants were asked about their thoughts on the concept of exercise addiction and whether they felt that it applied to them. Rather than provide participants with a formal medical-model definition of exercise addiction, it was preferred to understand the participants’ perspective. Finally, participants were invited to elaborate on the significance of exercise in their lives. On completion, participants were thanked for their time and signposted to support organisations or their GP if any concerns had been raised.

### Data analysis

Data analysis followed the stepped approach described by Braun and Clarke (2022). Manual transcription was used to facilitate the initial step of data familiarisation, followed by multiple readings of the transcripts with note taking to highlight initial observations of the data. Coding used an inductive approach to investigate the topics highlighted by participants and utilised NVivo software to enable consistency in the coding and to keep track of candidate themes. Coding was completed by the first author, supported by a reflexivity journal and routine meetings to discuss ideas around candidate themes with the second author, who acted as a ‘critical friend’ (Smith and McGannon, 2018). Themes were refined in an iterative process as a research team through discussion of reflexive interpretations of the data and the themes.

Although exercise is often considered gendered, the focus of the results below was to look beyond gendered differences and focus on shared experiences of the positive and negative aspects of exercise.

## Results

**Figure 1.**
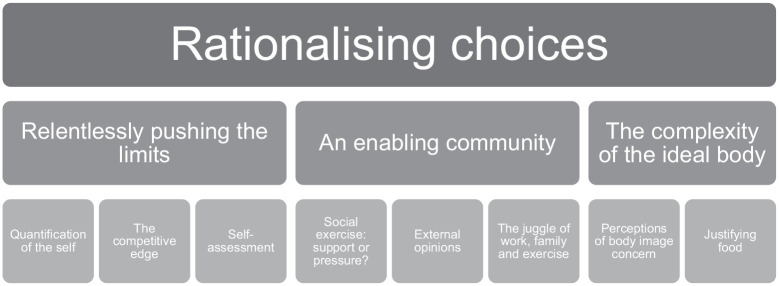
Thematic map.

Through the analysis of the interview data, three key themes were developed, each of which had sub-themes overarched by a transcending theme; a thematic map can be found in Figure 1. Together, these themes highlight some of the key features of the problematic experiences of frequent exercisers. These themes with their subthemes will now be described and illustrated with exemplar quotes.

### Theme 1 – relentlessly pushing the limits

This theme describes the feeling of always striving to improve, whether concerning one’s own personal best or in comparison to others. There was a sense of not being satisfied with one’s performance and of wanting to do more, be faster, be stronger or play better. This is fundamental to sporting progression and fitness improvement, however, some participants had negative reflections of this experience. Participants described the ways in which they relentlessly pushed the limits in terms of quantifying their exercise and other habits, the role of competitiveness in driving undesirable behaviours, and their self-assessment of their performance against their goals.

#### Quantification of the self

Most participants reported using a wearable fitness device often used in conjunction with smartphone apps, such as Strava, to quantify their behaviour. One participant, Richard, numbered each of his runs on Strava, as he chased the goal of his longest consecutive run streak:

I don’t know. My… my wife thinks I’m a bit obsessive about it, but I don’t think I am, I think, well, maybe I am, maybe that’s the first sign of being obsessive about something (Richard).

Richard talked in detail about the importance of his numbering system, and the weight of history of his running record on Strava. For him, this was an important aspect of his exercise routine to continue every day despite ongoing strain injuries in his leg and ankle, where ‘if I had a week off, it would probably all go, but I’m not having a week off because, you know, it’s [running] everyday.’ His comment above, however, hints at an alternative perspective, that of his wife, who may experience the devoted run-tracking differently to Richard. Other participants simply tracked and focussed on their personal best. For some, it was considered enjoyable and even helpful in preventing overtraining. However, self-quantification and the reduction of exercise to purely numerical results had negative consequences. Some participants discussed the feeling of being overly focussed on achieving certain times, leading to negative self-reflection.

I started doing a lot more running, and then I found my times started to kind of get better. And then obviously after a while you just plateau don’t you, and then I was like… never gonna get that dream of a sub 4 marathon or whatever it is… and I think sometimes that could…cause a bit of negativity (Alison).

Fitness watches and other wearables were important for many participants’ exercise habits. However, the framing of the use of the data was important to encourage routine and fitness improvements, whilst avoiding overtraining and not at the expense of other sources of feedback, such as injury, or emotional responses.

#### The competitive edge

Competition was a major motivator for exercise, particularly in team sports, running and triathlons. For many, competition was a crucial component for performing at one’s best. However, a competitive nature also led to some negativity:

There was definitely an internal voice going ‘Your body’s not good enough…you need to improve your body to … do that’ and … I’m quite competitive so … being in sports around people who are better than me, there are moments where I’m just like, well, ‘why can’t my body do that? That makes me sad’ (Zoe).

James expressed frustration at how his competitive nature could manifest as aggression when playing football (soccer).

If you’re losing quite a lot and you’re a competitive person, you don’t feel like you’re getting a fair rub of the green. You can try and put yourself about…try and find other ways to win…you lean into the dark arts of football, which I definitely have…grown that skill set as I’ve aged and slowed…and you come away a bit sort of annoyed at yourself for getting annoyed or reacting or snapping (James).

The perspectives of these frequent exercisers provide a mixed view on competitiveness, and its role in developing a problematic relationship with exercise. Competition was often linked to frustration, disappointment and a drive to push harder. For some participants, the competitive element added fun and enjoyment to the exercise, and an intrinsic part of their nature. Competitiveness is inextricably linked to sporting activities and has many benefits in achieving performance-related goals, however, the negative consequences should also be acknowledged.

#### Self-assessment

In addition to quantifying and comparing their fitness or athletic performance, many participants showed a strong emotional reaction to a self-assessment of their performance against their goals and expectations. For many, this led to a feeling of inadequacy and the sense that they would never be good enough.

there’s definitely negative self-talk around it. Like… not good enough, not fast enough… “why am I even here…Why am I even bothering to run with these people” ….or… “that older lady’s way in front of me” ….but then to be fair… I always end up…making myself feel bad when I then just make an excuse for myself. Like… “Oh my period’s coming” or “Oh I had my injuries setting me back and that’s why I’m a bit slow”, and now I feel bad for making that excuse…. I feel like it’s a cop-out almost (Sophie).

In summary, quantifying and tracking their performance and progress played a key role in pushing the limits for some exercisers. Competition led to feelings of inadequacy in some, in others to feelings of aggression. Feelings of a performance shortfall were also experienced when participants self-assessed their performance against their own goals, often feeling that they were not good enough. In setting, measuring and assessing fitness goals, participants demonstrated a relentless drive to improve and push their limits, from some perspectives this was seen as positive. In other participants there was a sense of never feeling that they can do enough, and the role of self-quantification, competition and negative self-assessment could be a factor in developing a more problematic relationship with exercise.

### Theme 2 – an enabling community

Participants described how the community around them had a substantial impact on promoting or hindering healthy exercise behaviours. Socialisation and feelings of encouragement contrasted with feelings of obligation, discussed in the theme ‘Social exercise: Support or pressure?’. Participants also talked about the external opinions on what classifies as healthy exercise behaviour in encouraging or hindering help-seeking. They also described the conflict and the protective elements provided by the juggle between work, family and exercise.

#### Social exercise: support or pressure?

The social aspects of sports and exercise can have a meaningful impact on one’s sense of self and place within a community. This can be an effective way to connect with existing friends and colleagues. However, for some exercisers, the social environment led to feelings of social pressure and a desire to avoid disappointing others.

I guess when I played as a team, there was a sense of…commitment to team and that was why you did it…I think actually now it’s a bit more freeing in the sense that if I feel tired, I just don’t need to push myself. Whereas I think when I would have felt tired when I was playing, there was that voice saying “Oh you just need to keep pushing etc. etc. Your teammates will be working hard” (Caitlin).

For many of the younger participants in this sample, a problematic enabling community was experienced through social media. Many representations of health and fitness viewed online were seen to promote unrealistic body image and exercise approaches.

I actually try and stay off…social media platforms such as Instagram for this like reasoning … I wouldn’t be surprised if they could increase people’s susceptibility…for these behaviours to become problematic. I think the very like binary messaging of exercise is good, do as much as you can…there was a lot of sort of supercharged, very intense sort of approaches (Louise).

Although nearly all participants talked about the social benefits they received from exercise, there was also a sense from some who felt obliged to exercise or exert themselves further than they would have liked because of social pressures to participate and perform.

#### External opinions

The interviews indicated that having others around can be a motivator and safeguard when exercising, and the participants often noted the importance of external opinions in preventing them from over-exercising. Naomi, who had disordered eating as a teen, discussed the impact of her parents finding out about her exercise habits, and how this was a turning point, providing insight into her behaviour.

I was trying not to let them know about, like how much I was exercising, and so when they found out about it and they were like “[Naomi] that’s crazy, like why? Why are you doing that? Like that’s not healthy”. I think I always had this perception that this is what healthy people do. I was obsessed with being ‘healthy’ …but when other people found out about what I was doing and said they were so shocked, that clicked a little bit, that this isn’t what someone who’s just trying to look after themselves is doing (Naomi).

On the other hand, the notion of exercise as a healthy habit can be a restricting factor in raising concerns with other people. When Charlie struggled with excessive exercise, he felt that there was no option to seek help because he felt he would not be understood by others:

I was doing too much exercise. I was just not feeling good about it and felt like I couldn’t stop because I couldn’t talk to anyone about it because it’s a healthy habit, so why would it be an issue (Charlie).

External opinions on what is considered healthy levels of exercise, therefore, could provide help or hindrance to the individual exerciser, either helping identify excessive behaviour or restricting open conversation.

#### The juggle of work, family and exercise

For many adults, it can be difficult to balance the demands on time between work life, family life and finding time for exercise. These conflicting demands were found in many participants, specifically with children and partners, highlighting feelings of conflict and the need to adjust both their family life and exercise routines.

The last year or so, I’ve been having a rough idea to train or run particular times and lengths and distances, but, adapting to life with a young child in the household means that you can’t always do what you want… I mean I’m holding this six-month-old bundle of joy and I’m sitting thinking I wish I could go for a run now (Paul).

For some participants, the conflicting demands of the juggle led to reduced exercise and felt like a protective factor against overexercising. Richard recalled a time when exercise felt problematic for him when working overseas. When the work placement was finished, he moved home, felt that work commitments took over, and his exercise behaviour adjusted back to normal. Alison also mentioned a significant lifestyle change, having a baby, as a turning point in her experience with exercise, changing her priorities to allow for more balance. Similarly, Andrew had a demanding job and a young family and felt that the difference between his exercise and those with exercise addiction was due to time availability. His life situation simply did not allow him to participate in any more exercise.

In summary, some participants found that significant life changes acted as a turning point away from problematic exercise because of the requirement to juggle other demands from work or family. Experiencing conflict and interference with everyday life is regularly listed as a criterion for addiction, however, these participants showed that a certain level of conflict was relatively common, even among those who did not perceive exercise as problematic. Furthermore, a lack of conflict in someone’s life could facilitate excessive focus on exercise.

### Theme 3 – the complexity of the ideal body

Many participants felt that weight management was a key benefit of exercise, whether viewed as weight loss or avoidance of weight gain. However, people’s perceptions of body image concerns varied depending on their motivations. Similarly, there were differing perceptions regarding the role of diet, with participants using exercise to justify the food they ate.

#### Perceptions of body image concern

Weight management was frequently mentioned in the interviews when discussing exercise motivations. When weight maintenance or avoidance of age-related weight gain was associated with health, it was viewed as a positive motivation. However, when people spoke about being motivated by aesthetic reasons, they felt less positive about weight control. For these participants, the idea of caring about their weight and body image was perceived as superficial.

I do like to have a good body image…it doesn’t sound too great to say…but…you know how there’s a male image of having like a good [body]…I do try and go for that (Marcus).

Naomi discussed an ongoing struggle to avoid thoughts linking exercise with the way she looked.

I’d love to say that I never thought about the impact it would have on how I look, that’s not true, but that’s not a motivational factor for me anymore … it’s the — it’s gonna make me more healthy, that it’s gonna make me stronger and I enjoy it and I get something out of it (Naomi).

Weight management was mentioned by most participants as a benefit of exercise; however, when related to physical appearance it was viewed as shallow, and this motivation was to be avoided if possible.

#### Justifying food

For many participants, the experience of exercise went together with their experience of eating habits. For some participants, exercise and diet worked together in a positive feedback loop, in which exercise led to better food choices. However, most participants who talked about food felt that their exercise helped justify the food they ate. Nevertheless, this was still seen as being relatively positive and enjoyable. They could eat the food they loved because they exercised.

I have like a really big appetite to be honest. I love eating so much and, you know, I’m a big foodie. But I guess if you eat a lot, in order to kind of maintain a certain size and body shape or whatever it also…requires that you exercise a lot (Louise).

For these participants, there appeared to be a need to justify the food they ate. However, this was not seen as a problematic relationship, it was principally regarding enjoyment. However, for Carlos, although he did not suggest that he had sought help or received a diagnosis of disordered eating, he felt that guilt related to his eating was more problematic.

I think I also have like, uh, issues with my eating, kind of thing, where I either, I’m very good at being sensible and kind of diet wise. But the moment I trip up, almost like a food addiction, I kind of go all in and I don’t stop even if I’m full, I’ll still snack afterwards and things like that, and I almost feel like running and cycling helps me feel a little bit less guilty for when those times do crop up (Carlos).

Many of these frequent exercisers used their exercise to justify their eating habits, which was experienced as a feeling of freedom to eat the types and volumes of food they wished. The level of guilt associated with food seemed to range from relatively benign to more problematic across a spectrum of exercise and eating habits. As expected, for participants who had experienced prior diagnoses of disordered eating, exercise had been perceived as a compensatory behaviour at that time.

### Transcending theme: Rationalising choices

Participants described problematic exercise behaviour in terms of relentlessly pushing the limits, tracking, competing and assessing their performance; the enabling community around the exerciser, which could either add pressure and encourage excessive behaviour; or provide support, alternative priorities and identify when exercise is no longer healthy and the complexity of the ideal body through weight loss motivations and food-exercise relationships. All themes demonstrated a mix of positive and negative experiences. Monitoring and recording fitness data can be motivating for some, but obsessive for others. Exercising with others can be sociable and improve performance but can also create a sense of obligation. Some participants felt joy and freedom from unrestricted eating, whereas others experienced guilt that they had to negate through exercise. Transcending these themes was the notion of rationalising choices.

For many of these participants, problematic exercise was facilitated through a process of rationalising their behaviour through a cost-benefit analysis. Reflecting this, while all participants acknowledged some negative consequences of their exercise, they believed that the positives outweighed the negatives. For Manpreet, the main negative consequence was tiredness which could creep into her childcare responsibilities, but this was rationalised by the idea that she felt she also lacked energy when she was not able to exercise.

Both Georgina and Paul described their exercise experiences as ‘overwhelmingly positive’, and how this positive experience outweighs the physical pain and numerous physical injuries caused by exercise.

I think the only negative thing, really, is the physical damage. What I have done to my body, because, you know, you do get injured. You know, and I’ve had a fractured pelvis, I’ve broken…fractures in various bones in my leg and my feet. So, it…it’s not all a bed of roses, exercise, but I think ultimately, the metabolic benefits outweigh these sort of short-term physical problems (Georgina).

When Zoe discussed the concept of exercise addiction, she talked about the idea that exercise can serve a larger purpose in a person’s life, and as long as it delivers those benefits, it is worth the cost.

I think, you know, like the same with any kind of addiction, like smoking or drinking and stuff, like you started it for a reason. And if you can’t get that reason into your routine without that thing, you’re never gonna give it up, like it’s not gonna work. So, I think that’s kind of where I sit. I think the exercise for me brings me that peace and that sort of the sense of self, that, unless I find it from somewhere else, I think I’m still gonna choose that addiction (Zoe).

These quotes demonstrate that although there are negative aspects to exercise, these frequent exercisers rationalised this against the positive outcomes that they also experienced. This rationalisation of the behaviour was shared even amongst the participants with seemingly more extreme exercise habits.

## Discussion

Much exercise addiction research to date is grounded in addiction criteria taken from the field of substance abuse, emphasising a dichotomised model of problematic behaviour. This qualitative study asked frequent exercisers about their own perceptions of problematic exercise. An inductive thematic analysis of these interviews generated three themes: ‘relentlessly pushing the limits’, ‘an enabling community’ and ‘the complexity of the ideal body’, alongside a transcending theme of ‘rationalising choices’. These findings demonstrated the various ways in which these frequent exercisers identified problematic behaviour and how they were balanced with the perceived benefits.

The first theme highlighted how many of these frequent exercisers had a clear focus on pushing their limits. This was manifested through quantification of the self, using fitness trackers to track progress and performance, feeling the consequences and benefits of competition and making a self-assessment of performance. Similar themes have been found in studies of self-proclaimed exercise addicts and female cyclists (Baker et al., 2023). Moreau et al. (2023) and Baker et al. (2023) also found useful and harmful aspects of pushing limits. In this current study, the same behaviour was found across a spectrum of exercise behaviours, regardless of self-identified addiction status, or frequency of exercise. This demonstrates that the use of wearable fitness technology, tracking devices and numerical-based goals can be useful from a performance perspective but should be used with caution. For example, technology can be used to highlight potential overtraining, but it should be used in conjunction with other aspects of feedback, both bodily sensations such as pain and from external sources such as personal trainers, coaches, friends and family.

The second theme, ‘An enabling community’, illustrates how those surrounding the exerciser play a key role in the perception of exercise. Consistent with prior research finding no significant differences in the risk of exercise addiction between those who play team sports and those who exercise alone (Lichtenstein et al., 2014), the participants in this study varied in their perceptions of solitary and social exercises. Many participants spoke of social exercise carrying additional peer pressure to perform, urging the exercisers to push beyond a level they are comfortable with, whereas others found that sociable exercise added an element of fun that protected them from problematic feelings. The role of the media in creating unrealistic expectations and promoting extreme and unachievable exercise routines was also discussed. The negative role of social media, in particular for ‘fitspiration’ (a visual means of fitness inspiration found on social media) has been found in prior research, which found that people who used ’fitspiration’ were at higher risk of eating disorders, psychological distress and exercise addiction (Raggatt et al., 2018).

The community around the exerciser can help identify when the exercise has become excessive, as demonstrated through the subtheme ‘External opinions’. This mirrors analysis of social media posts relating to compulsive exercise and eating disorders, whereby individuals sought out the opinions of others to ask ‘is my exercise healthy?’ (Hallward and Duncan, 2021). In contrast, other people’s opinions, particularly the perception that exercise must be healthy, seemed to restrict some exercisers from expressing their feelings or concerns as they felt that they would not be understood or taken seriously. This speaks to the wider public perception of the benefits of exercise, which are so often discussed and promoted. Understandably, much research has focussed on the promotion of exercise as a healthy behaviour (e.g. Wilson and Estabrooks, 2020), but the risks are rarely discussed. A more nuanced discussion around exercise, may help exercisers who are struggling with exercise to open up and feel more comfortable sharing their difficulties.

Embedded within this theme was also the role of juggling life demands. Conflict is often used as a classic diagnostic criterion for addiction, including exercise (DE Coverley Veale, 1987; Griffiths, 1997). However, in this study, many participants felt that the presence of conflict in their lives and the demands of multiple commitments allowed them to reprioritise and decide what was most important for them. Similarly, stress and significant life changes are usually associated with developing exercise addiction (Dinardi et al., 2021); however, some of the frequent exercisers in this study discussed life changes, such as the birth of a baby or moving countries, allowed them to re-assess and resulted in a move from problematic to healthy exercise behaviour. It could well be that a lack of other life priorities can facilitate more excessive behaviours. For many of the participants in this study, although there were signs of some conflict, this was not necessarily seen as problematic.

The final main theme, ‘the complexity of the ideal body’, reflected research related to problematic exercise and body image and its comorbidity with disordered eating. Many participants talked about weight loss or weight maintenance as a benefit of exercise. This supports the ‘creating the shape’ theme found by Cox and Orford (2004). However, compared with Cox and Orford’s research, this current study found a perception of being superficial and actively fighting the desire to be motivated by aesthetics. Excessive exercise is often used to counter binge eating (Hallward and Duncan, 2021). One participant in this study showed signs of this behaviour, although he did not suggest that he had sought help for disordered eating. Participants who showed no signs of eating distress also used exercise to justify eating. These participants framed this positively, allowing them to eat as much as they liked or indulge in treats which would otherwise make them feel guilty. Although using exercise to relieve eating-related guilt can clearly play a negative role in an individual’s life, this research highlights that there are times when this can be seen as positive.

Each theme generated in this study contained a mixture of positive and negative experiences. Interviews revealed a variety of perceptions regarding the problematic aspects of exercise. Many exercisers referred to experience with problematic exercise at some point in their lives and most used this experience to learn and moderate their behaviour. The key mechanism used by participants to justify their behaviour was rationalising choices. Even attitudes towards exercise that were perceived by the researcher team as more extreme (such as exercising to the point and beyond of breaking bones) were justified through a cost-benefit analysis. The frequent exercisers in this sample acknowledged the negative consequences of their exercise but stated that they were outweighed by the positives. Participants with a history of disordered eating stated that exercising was better and more important than not exercising. Those with more extreme exercise habits unrelated to eating disorders also felt that, despite broken bones or extreme pain, the benefits of exercise were worth it. The choice to continue exercise was then perceived as rational; however, it could be demonstrative of an overvaluation of reward and undervaluation of punishment or negative consequences.

Although the participants did not indicate compulsion to exercise, there were elements of some usual medical model diagnostic criteria for addiction across nearly all participants. For example, all exercisers in this sample expressed feelings of withdrawal when they were prevented from performing their usual exercise. Similarly, all participants had experienced some level of negative consequences due to exercise. However, this did not differentiate between varying amounts or attitudes towards exercise. This supports the idea of problematic exercising being on a continuum (Johnston et al., 2011). There was no clear boundary between exercisers’ perceptions of problematic and healthy behaviour. This research therefore suggests that the traditional methods of defining exercise addiction may not highlight areas which are genuinely problematic for the exercisers themselves. Dichotomising and pathologising may not reflect the lived reality of individuals who exercise. Instead, overfocusing on the benefits of exercise may facilitate problematic behaviour, as the benefits of exercise can be used to justify extreme amounts of exercise despite negative consequences. Perhaps a more balanced approach in discussions around exercise should also identify and acknowledge the possible negative consequences of exercise, rather than purely focussing on the benefits.

### Methodological limitations

There are some limitations of the present study that need to be considered. First, the self-selected interview sample may have had more preconceived notions of problematic exercise than the general exercising population. Although efforts were made to have a diverse sample in terms of demographics and chosen exercise, the group was relatively homogenous, with a predominantly white, highly educated sample; therefore, there may be some limitations in the generalisability of the results.

Further, due to participant time constraints, some interviews were not particularly long. However, the discussions that took place during the interviews were detailed, and participants were generous in their sharing of experiences. Longer interviews, or alternative methods of qualitative data collection such as focus groups may have resulted in further interesting and nuanced discussion.

The scope of this study did not allow for an in-depth review of differences in the experiences of primary and secondary exercise addiction. Primary exercise addiction occurs when exercise itself provides motivation, whereas secondary exercise addiction is driven principally by another condition such as disordered eating (Bamber et al., 2000). Some participants disclosed prior disordered eating diagnoses, which have been highlighted; however, the extent to which the experiences of problematic exercise within secondary exercise addiction differ should be explored in future research. It is expected and acknowledged that secondary exercise addiction typically is associated with significantly higher levels of psychological distress than primary exercise addiction (Bamber et al., 2000). The conclusions reached regarding choice and positives outweighing negatives may therefore not apply to this population and would warrant further investigation.

## Conclusion

This study aimed to explore the perception of problematic exercise behaviours through the experiences of frequent exercisers. The analysis highlighted three themes; ‘relentlessly pushing the limits’, ‘an enabling community’ and ‘the complexity of the ideal body’ transcended by the rationalisation of negative consequences of exercise through a cost-benefit analysis. This supports the notion of a continuum rather than a dichotomised approach to problematic exercise, together with an emphasis on perceptions of problems rather than objective definitions. Furthermore, while exercise has many benefits for both physical and mental health, an overfocus on these benefits by either the health care professional or the exercisers themselves may facilitate a rationalisation process which minimises costs and maximises benefits even in the presence of harm.
